# Whole-gene analysis of two groups of hepatitis B virus C/D inter-genotype recombinant strains isolated in Tibet, China

**DOI:** 10.1371/journal.pone.0179846

**Published:** 2017-06-27

**Authors:** Tiezhu Liu, Fuzhen Wang, Shuang Zhang, Feng Wang, Qingling Meng, Guomin Zhang, Fuqiang Cui, Dorji Dunzhu, Wenjiao Yin, Shengli Bi, Liping Shen

**Affiliations:** 1 National Institute for Viral Disease Control and Prevention, Chinese Center for Disease Control and Prevention, Beijing, China; 2 National Immunization Program, Chinese Center for Disease Control and Prevention, Beijing, China; 3 Tibetan Center for Disease Control and Prevention, Lhasa, Tibet, China; University of Cincinnati College of Medicine, UNITED STATES

## Abstract

Tibet is a highly hepatitis B virus (HBV) endemic area. Two types of C/D recombinant HBV are commonly isolated in Tibet and have been previously described. In an effort to better understand the molecular characteristic of these C/D recombinant strains from Tibet, we undertook a multistage random sampling project to collect HBsAg positive samples. Molecular epidemiological and bio-informational technologies were used to analyze the characteristics of the sequences found in this study. There were 60 samples enrolled in the survey, and we obtained 19 whole-genome sequences. 19 samples were all C/D recombinant, and could be divided into two sub-types named C/D1 and C/D2 according to the differences in the location of the recombinant breakpoint. The recombination breakpoint of the 10 strains belonging to the C/D1 sub-type was located at nt750, while the 9 stains belonging to C/D2 had their recombination break point at nt1530. According to whole-genome sequence analysis, the 19 identified strains belong to genotype C, but the nucleotide distance was more than 5% between the 19 strains and sub-genotypes C1 to C15. The distance between C/D1with C2 was 5.8±2.1%, while the distance between C/D2 with C2 was 6.4±2.1%. The parental strain was most likely sub-genotype C2. C/D1 strains were all collected in the middle and northern areas of Tibet including Lhasa, Linzhi and Ali, while C/D2 was predominant in Shannan in southern Tibet. This indicates that the two recombinant genotypes are regionally distributed in Tibet. These results provide important information for the study of special HBV recombination events, gene features, virus evolution, and the control and prevention policy of HBV in Tibet.

## Introduction

Hepatitis B virus (HBV) infection is a serious health problem and represents a substantial disease burden in China. During 2006, a nationwide hepatitis B investigation was carried out in China. It showed that 7.2% of the total population were HBV carriers testing positive for hepatitis B surface antigen (HBsAg) [1]. Carriers of HBV are at increased risk for developing cirrhosis and hepatocellular carcinoma (HCC) [2].

HBV genotypes/sub-genotypes may play an important role in causing different disease progression profiles during chronic hepatitis B infection. Genotype has been shown to have significant impact on clinical outcome, disease prognosis, and response to antiviral treatment [3, 4, 5]. To date, eight confirmed genotypes (A to H), two tentative genotypes (I and J) and more than forty sub-genotypes of HBV have been reported around the world [6]. HBV genotypes and sub-genotypes vary in geographical distribution. It has been reported that genotype A and D are found worldwide; while genotypes B and C are found most commonly in East and Southeast Asia; genotype E prevails in Africa; genotype F in native Americans; genotype G is found mainly in Europe and the USA, and genotype H is predominantly isolated in Mexico [7].

Strain recombination is a common phenomenon in HBV. It was suggested that recombination events are relatively frequent and play a significant role in HBV evolution. Meanwhile, the principle cause of recombination is co-infection within a host with multiple genotypes or sub-genotypes of HBV [8]. More than 30 recombinant strains have been reported in recent years, including recombinant forms from HBV genotypes A and D [9], B and C [10], B and G [11], C and D [12,13], and F and G [14] in several countries.

Previous studies carried out in China have shown that genotype B predominates in most of the southern areas; C predominates in the northern and middle areas; while D infection is relatively common in Xinjiang Uygur Autonomous Region[15]. Recently, more and more HBV recombinants have been isolated in China, including A/B[16] and A/C/G[17], and C/D recombinant has been found to be dominant in the Qinghai-Tibet plateau[12, 13]. The most important and prototypical recombinant strain is HBV C/D recombinant which has been identified in south-western China. Moreover, this same strain was identified in our previous survey, where it was reported that more than 95% of Tibetan HBV infections were with the HBV C/D recombinant strain [12].

After more than 20 years of hepatitis B vaccination, the HBsAg prevalence has reduced significantly in China. However, HBV is still endemic in Tibet because of its special geographic location and high baseline of HBV infection. As such, it is an interesting region for HBV strain identification and study. It has been reported that more than 15% of the Tibetan population are HBsAg positive [18]. A large proportion of people were infected with the HBV C/D hybrid strain. However, the recombinant characteristics and clinical significance of this strain have not been studied further. Therefore, to understand the genetic characteristics of the HBV C/D strains and their distribution and impact in Tibet must be evaluated to aid in programs for treatment and prevention.

In this survey, 19 whole gene sequences of HBV C/D recombinants were obtained from five areas in Tibet including Lhasa, Shannan, Linzhi, Ali and Rikaze. Two types of C/D recombinants have been analyzed and compared with other genotypes and previous findings improving our understanding of this novel hybrid strain.

## Materials and methods

### Ethical approval

The survey protocol conformed to the ethical guidelines of the1975 Declaration of Helsinki and was approved by the Ethics Committee of the Chinese Center for Disease Control and Prevention. The purpose of the study and the right to information were explained to the participants by research staff. Written informed consent was obtained from each participant before the interview and venous blood collection.

### Subject sample collection

A total of 60 subjects who were HBsAg positive were enrolled in the study. Those subjects were collected from community-based populations from five areas of Tibet including Lhasa, Shannan, Linzhi, Ali and Rikaze using multistage random sampling. Firstly, one county was selected at random from five areas, respectively. Secondly, two villages were selected from every county. Thirdly, populations of 18–59 years old were selected from every village. Total 580 subjects were enrolled and 60 HBsAg positive samples were found. The date of this survey was 12/20/2014. Basic information including name, gender, birthday and address were recorded on the questionnaire prepared beforehand and 5 mL of venous blood was taken from each participant. The serum was separated and stored at −80°C until use, and repeated freezing and thawing was avoided. HBV infection markers including HBsAg, anti-hepatitis B core antibody(anti-HBc), hepatitis B e antigen (HBeAg) and anti-hepatitis B e antibody(anti-HBe) were detected by chemiluminescent assays (AXSYM; Abbott Laboratories, North Chicago, IL, USA).

### HBV DNA extraction, whole-genome amplification and sequencing

HBV DNA was extracted from 200μL serum using QIAamp DNA Blood Mini Kit(Qiagen, Hilden, Germany). Full-length HBV DNA(~3.2kb) was amplified by Nested PCR performed in seven fragments through two rounds. Fragments 1 to 9 were amplified using the primers listed in Table 1. Amplification for the first round of PCR of whole-genome was performed in 50 μL reaction volume containing 10 μL extracted DNA and 25 μL premix Taq polymerase for 45 cycles at 95°Cfor 40 seconds, 60°C for 1.5 minutes, 68°C for 3.6 minutes, while the second round of fragments from 2 to 7 were performed in a 50μL reaction volume containing 3 μL extracted DNA and 25 μL premix Taq polymerase for 30 cycles at 95°Cfor 20 seconds, 50°Cfor 50 seconds, 72°Cfor 1 minute. The amplification for the first round PCR of fragment 8 was performed in a 50 μL reaction volume containing 10 μL extracted DNA and 25uL premix Taq polymerase for 35 cycles at 94°C for 35 seconds, 54°C for 30seconds, 72°C for 1minute, while the second round of fragment 9 was performed in a 50 μL reaction volume containing 3 μL extracted DNA and 25 μL premix Taq polymerase for 35 cycles at 94°C for 30 seconds, 54°C for 30 seconds, 72°C for 1 minute. Sequencing was performed on an automated DNA sequencer ABI 3700(PE Applied Biosystem). The Seqman model of DNAstar software was used to assemble the seven fragments, and the correct nucleotide position of the complete HBV sequence was revised through alignment with the reference sequence in the GenBank database.

**Table 1:** Primers for nest-PCR.

Primer	Step	Position (nt)	Sequence of primer
(1)**LongFS**	First round	1799–1826	CTGCGCACCAGCACCATGCAACTTTTTC
(1)**LongRS**	First round	1774–1801	CAGACCAATTTATGCCTACAGCCTCCTA
(2)**1847FS**	Second round	1847–1867	TGTTCATGTCCCACTGTTCAA
(2)**2394RS**	Second round	2394–2408	GGCGAGGGAGTTCTT
(3)**2298FS**	Second round	2298–2315	GACCACCAAATGCCCCTAT
(3)**2933RS**	Second round	2933–2954	TCGGGAAAGAATCCCAGAGGAT
(4)**2821FS**	Second round	2821–2840	GGTCACCATATTCTTGGGAAC
(4)**272RS**	Second round	272–291	TGAGAGAAGTCCACCACGAGT
(5)**179FS**	Second round	179–197	CTAGGACCCCTGCTCGTGTT
(5)**704RS**	Second round	704–725	CGAACCACTGAACAAATGGCACT
(6)**599FS**	Second round	599–619	GTATTCCCATCCCATCATCCTG
(6)**1286RS**	Second round	1286–1305	GCTAGGAGTTCCGCAGTATGG
(7)**1175FS**	Second round	1175–1190	GCCAAGTGTTTGCTGA
(7)**1788RS**	Second round	1788–1802	GCCTACAGCCTCCTA
(8)**CoreF1**	First round	1608–1627	ATGGAGACCACCGTGAACGC
(8)**CoreR1**	First round	2471–2489	CCCAGTAAAGTTTCCCACC
(9)**CoreF2**	Second round	1697–1718	TGAGGCATACTTCAAAGACTGT
(9)**CoreR2**	Second round	2352–2372	GCAGCAAAGCCCAAAAGACCC

### Phylogenetic analysis

The full-length HBV sequences obtained were compared with representative sequences from the HBV genotypes(A–D,F,I) as well as with sequences representing 15 sub-genotypes from genotype C(C1–C15) retrieved from the GenBank database. Sequence alignments were carried out using MEGA software version 5 (www.megasoftware.net), and phylogenetic trees were constructed using the neighbor-joining method, while bootstrap resampling and reconstruction were carried out 5000 times to confirm the reliability of the phylogenetic tree analysis. Genetic distance and pairwise distance comparisons were also carried out using the MEGA software mentioned above.

### Recombination analysis

SimPlot software was used to detect recombination in genome sequences of HBV using boot-scanning analysis with the following parameters: a window size of 200 bp, a step size of 20bp, 500 bootstrap replicates, gap strip on and neighbor-joining analysis. The recombination detection was performed by considering nine sequences: one putative recombinant sequence, eight reference sequences from genotypes A, B, C, D, E, F, G, H. Each informative site supports one of eight possible phylogenetic relationships among the nine taxa. Contiguous sites suggest a single phylogeny were inferred to represent regions between recombination breakpoints. Boot-scanning and cluster analysis maximizing ×2 parameter was used to identify the breakpoints, and P values for the subsequent division of the sequence into genotypes were calculated using Fisher’s exact test.

### Nucleotide sequence accession numbers

The HBV whole-genome nucleotide sequences of the 19 C/D recombinant strains reported in this article have been submitted to the GenBank database, and assigned accession numbers between KX660674 and KX660690 (17 strains), and KU519422 and KU519423.

## Results

A total of 19 whole genome sequences of HBV C/D recombinant strains were successfully obtained from 60 HBV infected samples. 30 partial genomes which included the S and C regions were also obtained. Since the X region of HBV was not easy to be amplified, which limit us to obtain more whole genome sequences. The name of those 19 whole-genome sequences are Tibet-Lhasa-1, Tibet-Shannan-1 to Tibet-Shannan-6, Tibet-Rikaze-1 to Tibet-Rikaze-5, Tibet-Linzhi-1 to Tibet-Linzhi-2, Tibet-Ali-1 to Tibet-Ali-5. The average age of those individuals infected with these strains was 36±5 years, of those 13(68.40%) were from male and 6(31.60%) from female participants (Table 2).

**Table 2:** Basic information of 19 HBV whole-genome sequences obtained from Tibet.

Area	Numbers of samples	Age(years)	Gender	
			Male	Female
**Lhasa**	1	45	1	0
**Shannan**	6	32±6	4	2
**Rikaze**	5	34±4	3	2
**Linzhi**	2	28±3	2	0
**Ali**	5	41±2	3	2
**Total**	19	36±5	13	6

### Phylogenetic analysis

**Fig 1:**
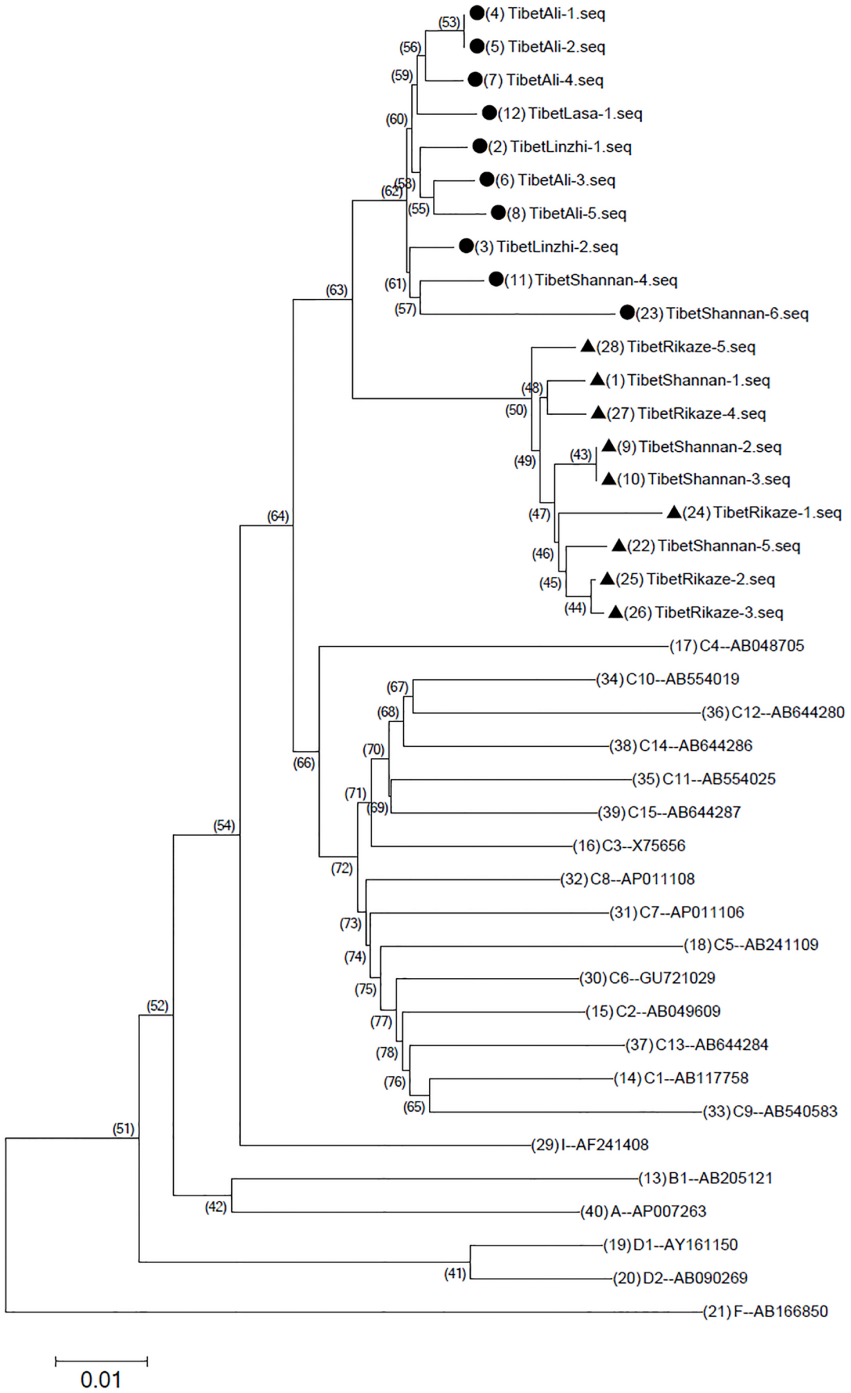
Phylogenetic analysis construct by whole-genome sequences of 19 HBV detected in this study compared with reference strains representing genotypes A,B,C,D,F,I.

Phylogenetic analysis based on the whole genome sequences suggested that the 19 HBV C/D recombinant strains are closely related (95.7%–100% homology). It was suggested by phylogenetic tree analysis that these strains belong to HBV genotype C. As shown in Fig 1, it is obvious that the whole genome sequences of these 19 samples are in two closely related clusters. In total, 10 strains are in one cluster (indicated by black dots) which includes Tibet-Ali-1-Tibet-Ali-5, Tibet-Lhasa-1, Tibet-Linzhi-1, Tibet-Linzhi-2, Tibet-Shannan-4, Tibet-Shannan-6. They were classified as HBV C/D1 recombinants. The other 9 stains formed another cluster (indicated by black triangles), which includes Tibet-Rikaze-1 to Tibet-Rikaze-5, Tibet-Shannan-1 to Tibet-Shannan-3 and Tibet-Shannan-5. They were classified as HBV C/D2 recombinants.

### Identification of the recombination sites

**Fig 2:**
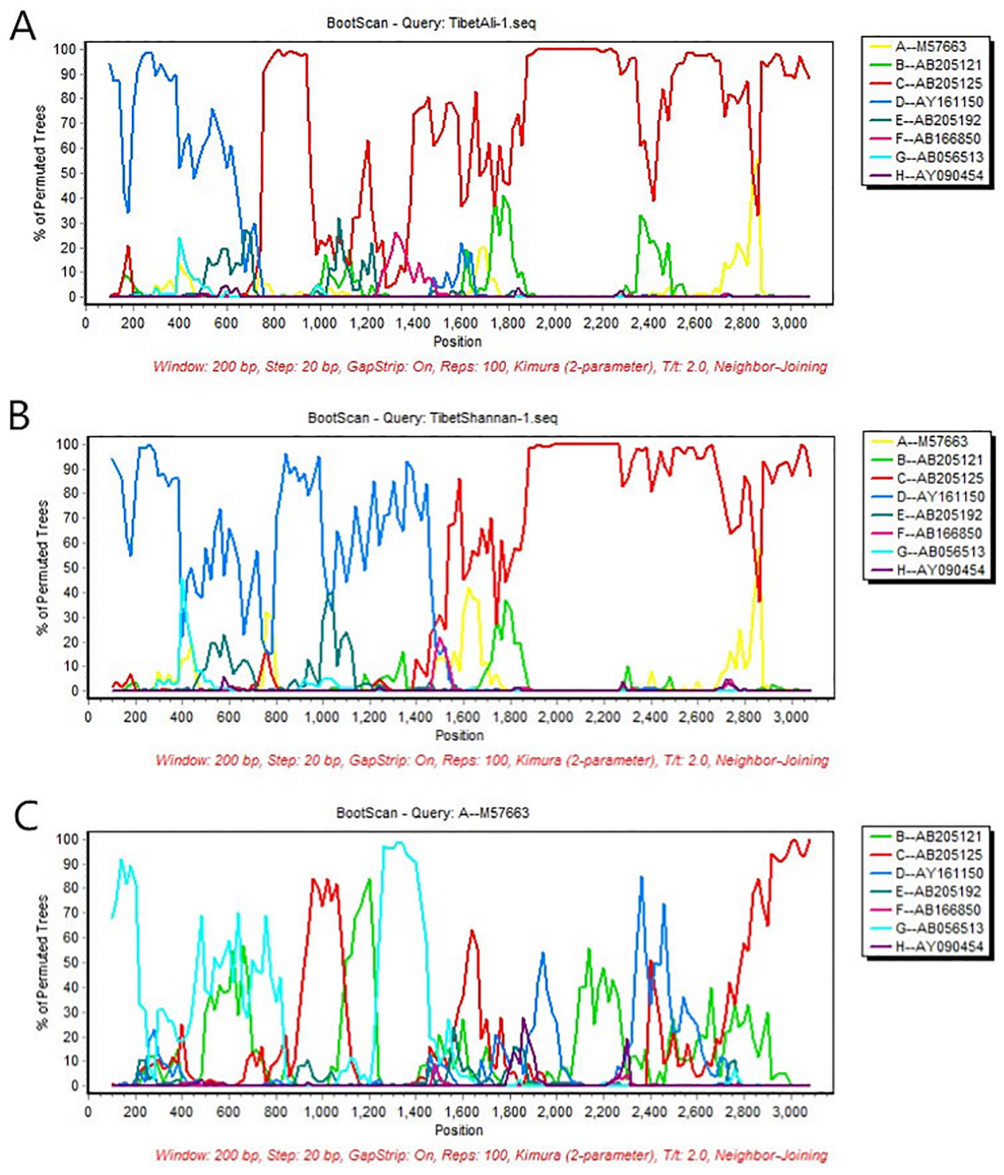
The results of bootscan analysis demonstrate the recombination of genotypes C and D. As shown in part A, TibetAli-1, which represents the C/D1 recombinant, was compared with eight representative HBV genotypes—A, B, C, D, E, F, G and H (GenBank accessions: M57663, AB205121, AB205125, AY161150, AB205192, AB166850, AV058513, AY090454, respectively). The recombinant position is at nt 750. As shown in part B, TibetShannan-1 represents C/D2; the recombinant position is at nt 1530bp.As shown in part C, genotype A represents the non-recombinant strain.

Determination of the breakpoints for genomic recombination in the 19 whole genome samples was assigned using the Simplot program. This analysis showed recombination between genotype C and D. Boot-scanning results suggested that in the genome of sample Tibet-Ali-1, which represented the C/D1 group, nt 0 to 750 were closely related to genotype D, whereas nt 751 to 3200 were closely related to genotype C. The breakpoint of C/D1 was located at nt 750 (Fig 2A). Meanwhile, boot-scanning results suggested that in the genomes of the sample Tibet-Shannan-1, which represented the C/D2 group, the nt 0 to 1530 were closely related to genotype D, whereas nt 1531 to 3200 were closely related to genotype C. The breakpoint of C/D2 was located at nt 1530(Fig 2B). Furthermore, all of the strains within these two clusters were considered C/D recombinant.

### Nucleotide sequence distance analysis

Comparing the nucleotide sequence distance of 10 C/D1 and 9 C/D2 strains with other reference genotypes and sub-genotypes, showed that the nucleotide distance to subtypes C1-C15 were all over 5%, and over 8% to the other genotypes (A, B, D, E, F, G and H). The distance between C/D1 and C/D2 were 1.6±0.3. The distance between C/D1 with C1 and C2 were 5.9±1.6% and 5.8±2.1%, the distance between C/D2 with C1 and C2 were 6.6±1.6% and 6.4±2.1% (Table 3). The distance values are all over the 4% divergence range and out of the current definition of a sub-genotype [6, 19].

**Table 3:** Nucleotide distance (%) among the two types of C/D recombinant strains and other reference genotype and subgenotype strains.

**Genotype**	**C/D1**	**C/D2**	**C1**	**C2**	**C3**	**C4**	**C5**
**C/D1**							
**C/D2**	1.6±0.3						
**C1**	5.9±1.6	6.6±1.6					
**C2**	5.8±2.1	6.4±2.1	4.9±1.6				
**C3**	6.7±2.3	7.3±0.8	5.0±1.7	6.0±2.4			
**C4**	6.7±2.4	7.4±0.2	6.8±0.7	7.8±1.3	6.4±0.3		
**C5**	5.6±2.4	6.2±2.1	5.5±1.5	6.6±0.7	6.0±2.2	8.2±2.3	

(Note: The reference genotype strains used in the table are similar to those used in Fig 1. C/D1 and C/D2 were compared with all reference strains; in order to save space, only C1–C5 were shown in this table.)

## Discussion

The sequencing and analysis of full-length genomes is crucial in determining the genotypes and sub-genotypes of novel HBV isolates. Partial genomes analysis may lead to uncertain classifications or missed recombinant patterns. It is valuable to study the whole genome of C/D recombinants to determine the nature of and site of valid recombination events. In this survey, 19 whole genome sequences for C/D recombinant strains were obtained. Furthermore, 30 partial genomes which included the S and C regions were also obtained. The preliminary analysis of those 30 partial genomes showed that they were all C/D recombinant strains.

It was reported that more than 60% of HBV recombinant isolates have their breakpoint between nucleotides 1640 and 1900 (located in the core region), recombinant strains with breakpoints in the *S* gene have also been identified [8]. In our study the breakpoints of C/D1 and C/D2 have been identified. The breakpoint of C/D1 was at nt750 located within the *S* gene, and breakpoint of C/D2 was at nt1530 located within the X region, respectively. The results were similar to Zhou’s reports in 2011, but in Zhou’s report they were unable to determine the breakpoint but did identify the possible recombinant region [13].

The preliminary results show that the area distribution of the 10 C/D1 strains was in the Ali, Linzhi, Shannan and Lhasa regions, while the 9 C/D2 strains were found in Shannan and Rikaze. It is interesting that all five samples from Rikaze were classified as C/D2, while the five whole genome sequences from Ali were all C/D1. It reinforces the geographical distribution of these two types of C/D recombinant strains, suggesting that they may have significant differences in evolution. This, however, needs to be confirmed by a much larger survey. No similar surveys about the geographical distributions of C/D1 and C/D2 in Tibet have been reported before.

The geographical distribution and origin of the HBV C/D recombinant is still unknown. It was reported that HBV C/D recombinant strains are not only prevalent in the Qinghai-Tibet plateau but have also been identified in Gansu and Ningxia provinces located near Tibet. However, most of the C/D recombinant strains were distributed in the Qinghai-Tibet plateau[12, 13].There are two reasons why this may be the case: Firstly, genotype D HBV is the most prevalent strain in India [16], while genotype C HBV is the predominant genotype in Southeast Asia and China [6,15]. The Qinghai-Tibet plateau is located between these two regions. This would provide a great opportunity for co-infection with both subtypes and thus the perfect opportunity for viral recombination. Secondly, the Qinghai-Tibet Plateau is a mountainous area, and visits from outsiders is uncommon because of its high altitude and the special religious considerations of the area. Tibetans were prevented from contact with the outside world as a result of these regional and cultural environmental conditions. It may explain why the C/D recombinant strains have emerged and flourished almost exclusively in this area. More studies are needed to determine whether C/D recombinant strains can adapt to the special genetic background and highland environment of Tibet.

HBV recombination is common in many areas of the world. Recently, some classical recombinant strains have been grouped as novel genotypes, including the African sub-genotype D8, which has been found to be a D/E recombinant [19], while in South-East Asia, HBV genotype I is a complex recombinant strain with a genotype C backbone [20, 21]. Recombination is also a source of misclassification of HBV and in many cases, recombinant strains have been wrongly introduced as new genotypes or sub-genotypes [22]. According to the traditional definition of HBV genotyping, HBV “sub genotype” is defined on the basis of a >4% and <8% divergence in complete nucleotide sequence with high phylogenetic bootstrap support [7]. However, some reports suggested that recombinant strains couldn’t be classified as a new genotype or sub-genotype even if their nucleotide differences exceed 8%. The “backbone” of genotype and other factors should also be taken into consideration. C/D recombinant strains are highly prevalent in the Qinghai-Tibet plateau, and it has been suggested that these strains may also be present in the nearby areas including Nepal, Budan and even India. Even if the nucleotide difference between C/D and other genotypes were over 8%, it is not appropriate to classify C/D recombinants as a new genotype or sub-genotype. A new term “recombino-subgenotype” was introduced to define the recombination. We proposed to classify the two types of C/D strains as prototypes of a new “recombino- subgenotype”. [23].

There are three limitations in this survey. Firstly, in order to describe the geographical distribution, more samples should be enrolled. Secondly, some index of liver functions and liver damage should be detected. Thirdly, not all seven areas in Tibet but just five areas were enrolled. Those limitations would be considered in the future survey. Further investigation should be carried out to collect more samples of C/D recombinant strains. Furthermore, because of the special geographical and relative poor hygiene conditions, HBV C/D recombinant strains may pose a significant public health risk. The clinical significance, future outcomes and disease burden have not yet been fully studied, but should be a priority in the region.

## Supporting information

S1 Table S1 Table shows the serum markers and DNA level of the 60 HBV infected samples. (XLSX)
